# Venous Thromboembolism Occurrence in the Setting of Nexplanon Insertion with Multiple Risk Factors: A Case Report

**DOI:** 10.3390/healthcare13202563

**Published:** 2025-10-11

**Authors:** Jennifer Chin, Sarah Taga

**Affiliations:** 1 Department of Obstetrics and Gynecology, University of Washington, P.O. Box 356460, 1959 NE Pacific Street, Seattle, WA 98195, USA; 2 School of Medicine, University of Hawaii, 651 Ilalo St., Honolulu, HI 96813, USA

**Keywords:** contraception, postpartum, subdermal implant, venous thromboembolism

## Abstract

Postpartum patients experience a 60-fold increased risk for venous thromboembolism (VTE). We present a postpartum patient with severe pre-eclampsia, gestational diabetes, and a recent Cesarean delivery, who was diagnosed with a VTE hours after a Nexplanon insertion on venous duplex ultrasound. She was started on anticoagulation, had the Nexplanon removed, and recovered well. This case highlights the importance of clinical suspicion for VTE in the postpartum period, presenting a postpartum VTE coinciding with a subdermal implant placement.

## 1. Introduction

We present a rare occurrence of a deep vein thrombosis (DVT) in a postpartum patient less than 24 h after Nexplanon insertion. Nexplanon is a subdermal, hormone-releasing contraceptive implant that is placed just under the skin of the inner, non-dominant upper arm. After insertion, the Nexplanon implant maintains contraceptive effectiveness for a duration of 5 years following insertion [1]. In the immediate postpartum period, it is not associated with an increased rate of venous thromboembolism events (VTEs) such as DVT or pulmonary embolisms (PE) [2]. However, there is limited evidence demonstrating an increased risk overall, with a recent study finding an odds ratio of 1.40 (95% CI 0.58–3.38, *p* = 0.450) when comparing DVT occurrence for non-hormonal contraception vs. Nexplanon [3].

This case report will cover an occurrence of a postpartum DVT event after receiving a Nexplanon implant. This is important as it highlights the need for a high index of suspicion for a postpartum DVT in a patient with multiple risk factors.

## 2. Case Report

A 21-year-old woman, gravida 3, para 2012, gave birth via her second Cesarean section under general anesthesia after multiple failed spinal attempts. Prenatally, this patient had a history of pre-eclampsia with severe features, vulvar varicose veins, genital herpes simplex virus (HSV), and diet-controlled gestational diabetes (GDMA1).

At the patient’s 1-week postpartum visit, she requested a Nexplanon implant for contraception at her next visit, which was placed 3 weeks postpartum. She was counseled on all other contraceptive options and, using shared decision making, opted for the Nexplanon. At the time of Nexplanon insertion, she had normal vital signs, was recovering well from her delivery, and had been exclusively breastfeeding for 3 weeks. The Nexplanon was inserted without complication. Later that evening, about 8 h after her appointment, the patient called complaining of arm pain, but denied any headache, vision changes, chest pain, dyspnea, or abdominal pain. Her arm became extremely swollen over two hours, and the pain started to radiate up her neck and down her left leg. It was recommended to the patient that she present to the nearest emergency department (ED).

At the patient’s ED visit, the Nexplanon insertion site was noted to have mild swelling, oozing, and bruising but no significant erythema. The implant was still palpable under the skin. The patient exhibited normal motor function of their left arm but experienced decreased sensation to light touch at the medial aspect of her left hand. Upper extremity venous Doppler ultrasound noted a nonocclusive deep venous thrombosis within the left internal jugular and proximal subclavian veins. Other imaging, including chest X-ray, and lab workup, including complete blood count, complete metabolic panel, and coagulation panel, were all within normal limits. The patient’s prior intravenous catheter sites appeared normal, and she reported that she had been ambulating without difficulty after delivery and denied an excessive amount of bleeding. Obstetrics and gynecology were consulted, and the patient was prescribed Lovenox, 100 mg, to be taken every 12 h. The patient was discharged with a referral to hematology.

The next day, the patient was informed that it would be safe to continue with the Nexplanon as the DVT clot was most likely present prior to the Nexplanon insertion; however, she was also given the option for removal if she felt most comfortable with that plan. She elected to have it removed by her primary care provider the following day.

Hematology agreed that the DVT was not likely caused by the Nexplanon implant, given that symptoms arose only a few hours after the implant was inserted. A DVT caused by a contraceptive method would typically present much later after initiation, on average, five weeks [4]. A diagnostic panel was performed of both acquired and hereditary disorders, leading to a potential hypercoagulable state. All tests came back negative, and the patient was instructed to continue Lovenox for three months, and to follow up with hematology soon after finishing with a repeat lupus panel and D-dimer. The timeline of these clinical events is shown in the Supplementary Materials.

The patient was seen back with hematology 3 months later. Repeat lupus tests came back negative, and D-dimer was undetectable. During this visit, the patient reported she was three months pregnant and planning to continue the pregnancy. A comprehensive plan was created to prevent recurrence throughout the patient’s pregnancy and postpartum period, which included 40 mg Lovenox daily antepartum, switching to heparin at 36–37 weeks of gestation, inducing labor at 39 weeks, then restarting Lovenox at 40 mg twice a day until 6 weeks postpartum.

## 3. Discussion

This case highlights the need for a high index of suspicion for diagnosis of VTE in the postpartum period, regardless of the choice of contraception. The risk for VTE increases during pregnancy by fivefold and in the three months after delivery by 60-fold, respectively [5].

Progestin-only forms of birth control are not well understood in their role in causing VTE. There is a broader discourse surrounding combined oral contraceptives (COCs). The estrogen component of COCs is known to increase the level of procoagulant factors (FVII, FIX, FX, FXII, and FXIII) and decrease the level of anticoagulant factors such as protein S and antithrombin. The significance of these effects seems to increase or decrease depending on the type of progestin in the combination [6].

In some comparative literature, progestin-only contraceptives have shown a lower risk of VTE in comparison to combined oral contraceptives (COCs). Looking specifically at recurrence, a study showed that COCs were associated with a 13.9% risk of VTE recurrence, while oral progestin-only contraceptives were associated with a 3.3% risk of recurrence [7]. Looking at primary VTE occurrence, progestogen-only therapy was associated with an increased risk when compared to non-use, with an odds ratio of 1.48 (95% CI 1.24–1.78) [8].

This patient also had many significant risk factors for VTE, including Cesarean section, multiparity, severe pre-eclampsia, GDMA1, and varicose veins. Cesarean sections and multiparity are two of the most significant risk factors for VTE occurrence, which increase risk by 2.1-fold and 1.6-fold, respectively [9,10]. Severe preeclampsia increases the risk for VTE in the postpartum period by 2.45-fold [11]. GDMA1 and varicose veins are also associated with increased risk of VTE and DVT with odds ratios of 1.40 (95% CI 1.36–1.43) and 1.17 (95% CI 1.07–1.29), respectively [12,13]. Although causality was not established in our case report, this is still an important clinical presentation for clinicians to be aware of, as this patient’s symptoms could have been dismissed as routine post-insertion discomfort. If this diagnosis had been missed, this patient could have suffered significant morbidity or potentially mortality.

Limitations of our case report include the multiple other risk factors this patient had that likely made her prone to developing a VTE regardless of the contraceptive method chosen at her postpartum visit. However, this case still highlights the need for an increased index of suspicion after any contraceptive initiation in patients with multiple risk factors.

There is limited literature examining the intersection of these risk factors and the specific role of progestin-only contraceptives in VTE risk. Further research is essential to support accurate, evidence-based counseling for patients seeking contraception in the postpartum period. Expanding this knowledge base will also enhance patient education on hormonal contraception more broadly, ultimately promoting safer and more informed reproductive healthcare decisions for all patients.

## 4. Conclusions

In the postpartum period, a high index of suspicion is required for VTE diagnosis. With the diagnosis of VTE in the postpartum period, anticoagulation is necessary both during the postpartum period and in subsequent pregnancies. The current literature offers limited insight into the VTE risk associated with progestin-only contraceptives, particularly subdermal implants like Nexplanon. This gap in knowledge underscores the need for further research to better define the safety profile of progestin-only methods. Expanding the evidence base will be essential to improving clinical counseling and ensuring patients can make informed decisions about contraception during high-risk periods such as the postpartum phase.

## Data Availability

The original contributions presented in this study are included in the article. Further inquiries can be directed to the corresponding authors.

## Supplementary Materials

The following supporting information can be downloaded at: https://www.mdpi.com/article/10.3390/healthcare13202563/s1, Table of Patient Risk Factors and Clinical Events.

## Abbreviations

The following abbreviations are used in this manuscript:

VTE	Venous thromboembolism
DVT	Deep vein thrombosis
PE	Pulmonary embolism
COC	Combined oral contraceptive
HSV	Herpes simplex virus
GDMA	Diet-controlled gestational diabetes
ED	Emergency department
