# Supplementary material for: Venous Thromboembolism Occurrence in the Setting of Nexplanon Insertion with Multiple Risk Factors: A Case Report
Source: Healthcare (Basel). 2025 Oct 11;13(20):2563. doi: 10.3390/healthcare13202563 (PMC12562538; doi:10.3390/healthcare13202563)
Supplement: Supplementary file 1 [file healthcare-13-02563-s001.zip › healthcare-3823916-supplementary.pdf]

Table of Patient Risk Factors and Clinical Events

**Risk Factors**	
Severe pre-eclampsia	
Gestational diabetes	
Vulvar varicose veins	
Cesarean delivery	
General anesthesia	
Multiparity	
**Clinical Events**	**Days from Nexplanon Insertion**
Cesarean delivery	21 days prior
Nexplanon insertion	0
DVT diagnosis	0
Initiation of Lovenox	0
Nexplanon removal	2
Discontinuation of Lovenox	93
